# Diagnostic Performance of 18-Fluorodeoxyglucose Positron-Emission Tomography-Computed Tomography (18F-FDG PET-CT) in the Detection of Autosomal Dominant Polycystic Kidney Disease Cyst Infections: A Meta-Analysis

**DOI:** 10.7759/cureus.70863

**Published:** 2024-10-04

**Authors:** Deepak Chandramohan, Prathap Kumar Simhadri, Prabhat Singh, Jyotsna Gummadi, Rachna Valvani, Nihar Jena, Sreekant Avula

**Affiliations:** 1 Nephrology, University of Alabama at Birmingham, Birmingham, USA; 2 Nephrology, AdventHealth, Daytona Beach, USA; 3 Nephrology, Christus Spohn Hospital, Corpus Christi, USA; 4 Internal Medicine, MedStar Franklin Square Medical Center, Baltimore, USA; 5 Internal Medicine and Geriatrics, North Alabama Medical Center, Florence, USA; 6 Cardiovascular Medicine, Wayne State University, Pontiac, USA; 7 Diabetes, Endocrinology, and Metabolism, University of Minnesota, Minneapolis, USA

**Keywords:** 18fdg-pet, adpkd, cyst, infection, kidney

## Abstract

Diagnosing suspected renal or hepatic infections in autosomal dominant polycystic kidney disease (ADPKD) is difficult. Although 18-fluorodeoxyglucose positron-emission tomography-computed tomography (^18^F-FDG PET-CT) is recommended to aid in the diagnosis, there is no consensus about its diagnostic accuracy. We aimed to investigate its diagnostic performance. To further assess this, we performed a meta-analysis. A comprehensive literature search screening of PubMed/MEDLINE, Embase, and Cochrane library databases through February 2024 was performed. Pooled sensitivity, specificity, positive predictive value (PPV), negative predictive value (NPV), and C-reactive protein (CRP) were estimated using the random effects model. Heterogeneity between studies was estimated using Cochran Q and I2 statistics. A total of seven studies were included in the final analysis. The pooled sensitivity of ^18^F-FDG PET-CT in diagnosing kidney and hepatic cyst infection was 82.6% (95% CI: 73.8-88.9; I2 16.9%), specificity was 77.6% (95% CI: 66.7-85.7; I2 15.6%), PPV was 79.4% (95% CI: 62.4-89.9; I2 62.6%), and NPV was 81.3% (95% CI: 72.7-87.7; I2 0%). The mean CRP was 244.2 mg/L (95% CI: 209.1-279.1; I2 66%). The results showed that ^18^F-FDG PET-CT demonstrated excellent pooled diagnostic performance in diagnosing renal and hepatic cyst infections in ADPKD.

## Introduction and background

Autosomal dominant polycystic kidney disease (ADPKD) is a prevalent hereditary renal disorder that affects around one per 500-1000 population. Numerous renal cyst formations characterize ADPKD, originating from different segments of the renal tubules, ultimately resulting in enlarged kidneys. These cysts contribute to several complications in patients with ADPKD, such as intracystic hemorrhage, obstruction, pain, gross hematuria, and, most significantly, cyst infections [1]. The occurrence of cyst infections is a severe complication that necessitates hospitalization and treatment with intravenous antibiotics. Some cases may require other invasive procedures, such as excision. Since ADPKD results in extra-renal manifestations such as hepatic cysts, infected hepatic cysts can also occur [2]. Diagnosing cyst infections is challenging, and the common conventional imaging modalities have inherent limitations. The gold standard of diagnosis is the aspiration of the cyst and demonstrating the presence of neutrophils or bacteria with the subsequent culture of the aspirate, but this is not always obtainable due to failure to identify the location of the infection by routine imaging or the suspected focus of infection cannot be reached by percutaneous means [3]. Ultrasonography, computed tomography (CT), or magnetic resonance imaging (MRI) were noted to be unable to detect the infected cysts in 94%, 82%, and 60% of instances, respectively. Moreover, these conventional tests also carried a high rate of false negativity [1]. Currently, clinicians rely on clinical, biochemical, and imaging modalities for diagnosis, but it could only be reached in about 68% of cases suspected to have definite cyst infections [4].

Recently, 18-fluorodeoxyglucose positron-emission computed tomography, combined with computed tomography (^18^F-FDG PET-CT), has become a novel hybrid imaging technique in cyst infection diagnosis and found to be superior to CT and MRI [5]. 18-fluoro-2-deoxy-D-glucose (FDG), a glucose analog, is used due to its ability to be naturally taken up by cells, particularly those parts of the body with infection or inflammation [6]. Although ^18^F-FDG PET-CT is recommended for diagnosing renal and hepatic cyst infections, there is an overall limited knowledge about the efficacy of this modality due to the lack of literature available on the subject [5]. In this study, we performed a systematic review and meta-analysis to determine the diagnostic performance of ^18^F-FDG PET-CT in detecting renal and hepatic cyst infections.

## Review

Methods

Search Strategy

We conducted a literature search on the multiple electronic databases PubMed, Scopus, Web of Science, and Cochran Library from inception to March 2024. A combination of the following keywords was used in the search; "PET" OR "Positron-emission tomography" OR "CT" OR "Computed tomography" AND "Autosomal dominant polycystic kidney disease" OR "ADPKD" OR "Polycystic kidney disease" OR "Renal cyst infection" OR "Kidney cyst infection" OR "Liver cyst infection" OR "Hepatic cyst infection". In addition to these, we also searched the bibliographic sections of the retrieved studies to identify additional publications. The literature search strategy is presented in the Appendix.

Study Selection

The retrieved articles were examined separately by two authors (D.C and P.S). We initially conducted a screening process that involved the examination of all the titles and abstracts. Following the screening process, we reviewed full texts to assess the eligibility of a study using inclusion and exclusion criteria. Reviewer disputes were resolved by further discussion and consulting a third author, R.V. We included only the studies that had complete information. If studies had overlapping cohorts and patient data.

The meta-analyses of observational studies in epidemiology (MOOSE) checklist was followed and is listed under supplementary data [7]. The final articles were selected using the Preferred Reporting Items for Systematic Reviews and Meta-Analyses (PRISMA) guidelines [8]. The PRISMA checklist is shown in the Appendix.

The inclusion criteria are: (1) adults aged 18 years or older; (2) availability of information on ^18^F-FDG PET-CT; (3) studies published in English. The exclusion criteria are (1) studies reporting the diagnosis of infections other than cyst infections; (2) reviews, letters to the editor, case reports, and abstracts; (3) studies with less than ten patients; and (4) studies with missing information about the diagnostic performance of ^18^F-FDG PET-CT. The study protocol was registered in PROSPERO, CRD42024497973.

Data Abstraction

Two authors extracted the data (D.C and R.V) into a standardized form. The author's name, the country where the study was conducted, demographic information, sensitivity, specificity, and positive and negative predictive values of ^18^F-FDG PET-CT.

Quality Assessment

The quality of studies was assessed using the Quality Assessment of Diagnostic Accuracy Studies 2 (QUADAS-2). It assesses the risk of bias in studies using the domains: patient selection, the index test, the reference standard, flow, and training [9]. Two authors, D.C and P.S., independently performed the assessment. Visualization of the evaluation was done using Robvis [10].

Outcomes

The primary outcomes assessed were the pooled sensitivity, specificity, positive predictive value (PPV), and negative predictive value (NPV). The secondary outcome assessed was the pooled mean C-reactive protein (CRP) level.

Statistical Analysis

Continuous variables were expressed using mean ± standard deviation and categorical variables as percentages. The studies included in the analysis were selected as a random sample from a larger pool of studies. Therefore, we adopted the random effects model [11]. To determine pooled rates and their 95% confidence interval (95% CI), we utilized the inverse variance random effects DerSimonian-Laird method [12]. Forest plots were created to visualize the results of the statistical analyses [13].

The presence of heterogeneity was assessed using two different methodologies. Initially, we utilized the Cochran Q statistic to detect if the null hypothesis holds true that all the studies included in the analyses had the same effect size. If true, then the value of Q would be equivalent to the degrees of freedom, calculated by subtracting one from the number of studies. If the value of Q is more than the degrees of freedom, we will reject the null hypothesis [14]. The alpha threshold for this test is commonly set at 0.10 rather than 0.05 because the test lacks statistical power. When heterogeneity was detected by the Q statistic, we employed the I2 statistic. The I2 statistic measures and quantifies the proportion of variability in effect sizes that is not solely due to sampling error. The measured heterogeneity by I2 is quantified as low if values are <30%, moderate if values are between 31-60%, substantial if 61-75%, and considerable if >75% [15]. The statistical analyses were conducted using Comprehensive Meta-Analysis Software, version 4 (Biostat, Englewood, NJ, USA) [16].

Results

Search Results

**Figure 1:**
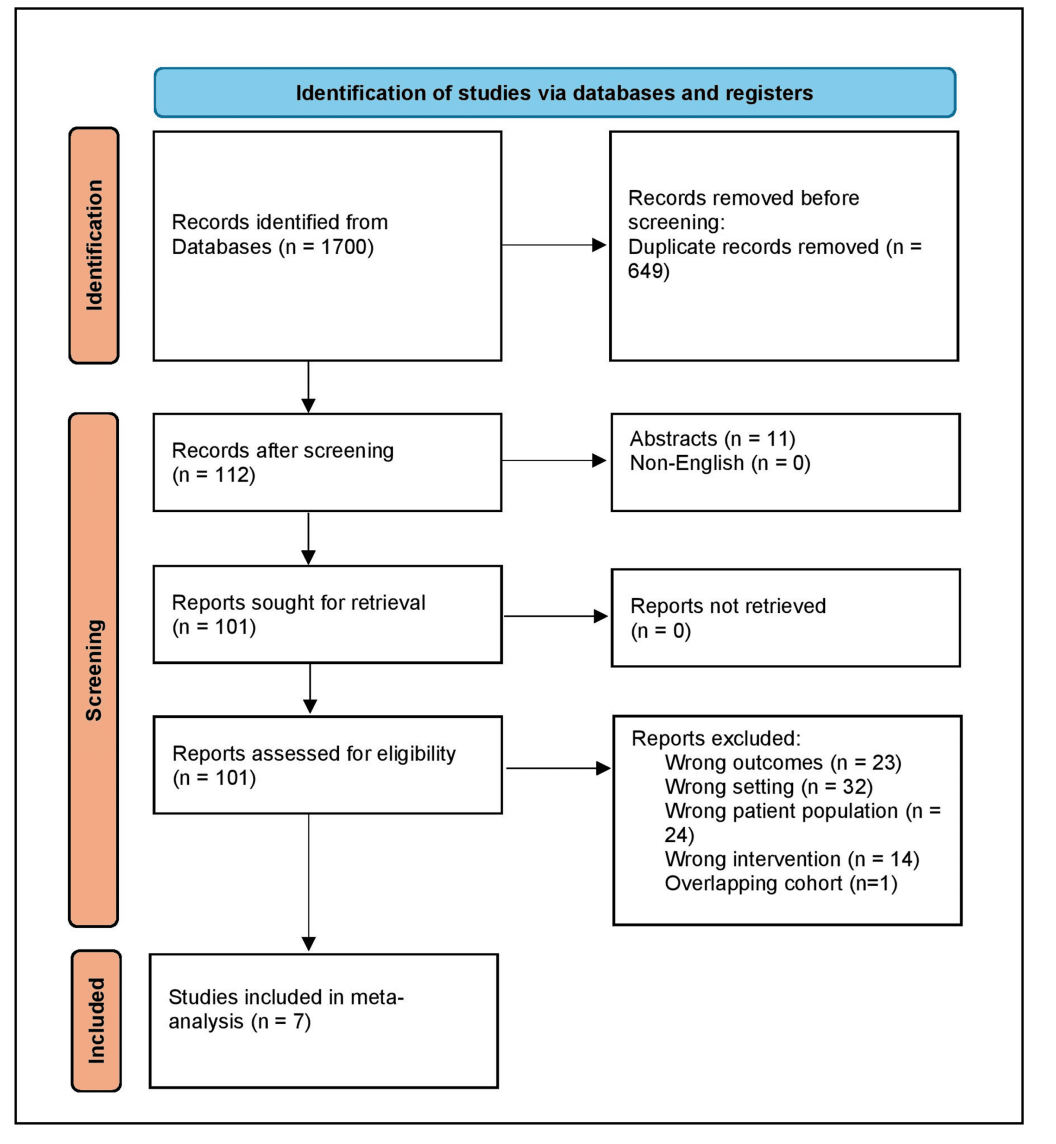
Study selection process according to the Preferred Reporting Items for Systematic Reviews and Meta-Analysis statement.

The search retrieved 1700 citations. After the exclusion of duplicates, screening was done to exclude case reports and review articles. Following the screening, the full texts of 101 articles were reviewed for their eligibility to be included in our study. Finally, seven studies met the inclusion and exclusion criteria and were included in our meta-analysis. A detailed flowchart of the study selection process is detailed in Figure 1. 

Study Characteristics

There were a total of seven studies included in the meta-analysis [17-23]. Five studies were retrospective cohort studies [17-19,22,23]. The study by Kwon et al. was a prospective study [21]. The study by Jouret et al. was a retrospective case series [20]. A summary of the included studies and their findings are shown in Tables 1, 2. 

**Table 1:** Summary of the included studies. CT: computed tomography; ^18^-FDG: fluorine-18 fluorodeoxyglucose; ^18^F-FDG PET-CT: 18-fluorodeoxyglucose positron-emission tomography-computed tomography; TKV: total kidney volume; HtTKV: height-adjusted total kidney volume.

Study	Year	Study type	Country	Total, n	ESRD, n	Outcomes
Balbo et al. [17]	2014	Retrospective, observational, single center	Brazil	18	6	Kidney cyst infection was high in patients with high total kidney volume (TKV) and height-adjusted total kidney volume (HtTKV). Liver cyst infection was higher in higher total liver volumes (TLV). Antibiotic modification was frequently required. Large cyst infections required drainage.
Bobot et al. [18]	2015	Retrospective, observational, single center	France	24	7	^18^F-FDG PET-CT was superior to CT. It is recommended to be performed within seven days of antibiotic initiation in patients with suspected cyst infection.
Demuynck et al. [19]	2023	Retrospective, observational, single center	Belgium	51	11	The study protocol was similar to Neuville et al. The study also validated the four-point scoring scale for suspected infections with an odds ratio of 6 for ≥3 points on the scale corresponding to a sensitivity of 64%.
Jouret et al. [20]	2011	Retrospective, observational, single-center case series	Belgium	27	2	^18^F-FDG PET-CT was able to detect kidney, liver cyst infections as well as other sources of infection in cases of fever of unknown origin compared to CT. In addition, there was abnormal uptake in patients who had cyst hemorrhage.
Kwon et al. [21]	2016	Prospective, observational, single center	Korea	17	9	^18^F-FDG PET-CT was superior to CT or MRI in detecting cyst infections. PET imaging was done 30 minutes after 18 FDG was injected. Other studies used longer wait times after ^18^-FDG injection.
Neuville et al. [22]	2021	Retrospective, observational, single center	Belgium	38	2	A four-point scoring scale was used to improve the visual assessment of ^18^F-FDG PET-CT and improve its diagnostic yield.
Pijl et al. [23]	2018	Retrospective, observational, single center	Netherlands	30	6	Extrarenal, extrahepatic inflammatory, and infections were also detected by ^18^F-FDG PET-CT. Other focus of infections was also detected.

**Table 2:** Studies showing the performance of 18-fluorodeoxyglucose positron-emission tomography-computed tomography (18F-FDG PET-CT). CRP: C-reactive protein; NR: nor reported; SD: standard deviation.

Study	Sensitivity %	Specificity %	Positive predictive value %	Negative predictive value %	CRP (mg/L), mean ± SD
Balbo et al. [17]	95	NR	NR	NR	258.9 ± 98
Bobot et al. [18]	77	100	100	77	193 ± 116
Demuynck et al. [19]	64	78	44	89	NR
Jouret et al. [20]	91	NR	85.7	NR	260 ± 14
Kwon et al. [21]	85.7	87.5	85.7	87.5	NR
Neuville et al. [22]	85.3	70.6	78.3	78.4	NR
Pijl et al. [23]	88.9	75	84.2	81.8	NR

Patient Characteristics

The total number of patients with suspected cyst infections was 203 and of these, the total number of end-stage renal disease (ESRD) patients was 43. The mean age was 56.6 years (95% CI: 53.4-59.8; I2 83%). Females were 50.5%.

Outcomes

**Figure 2:**
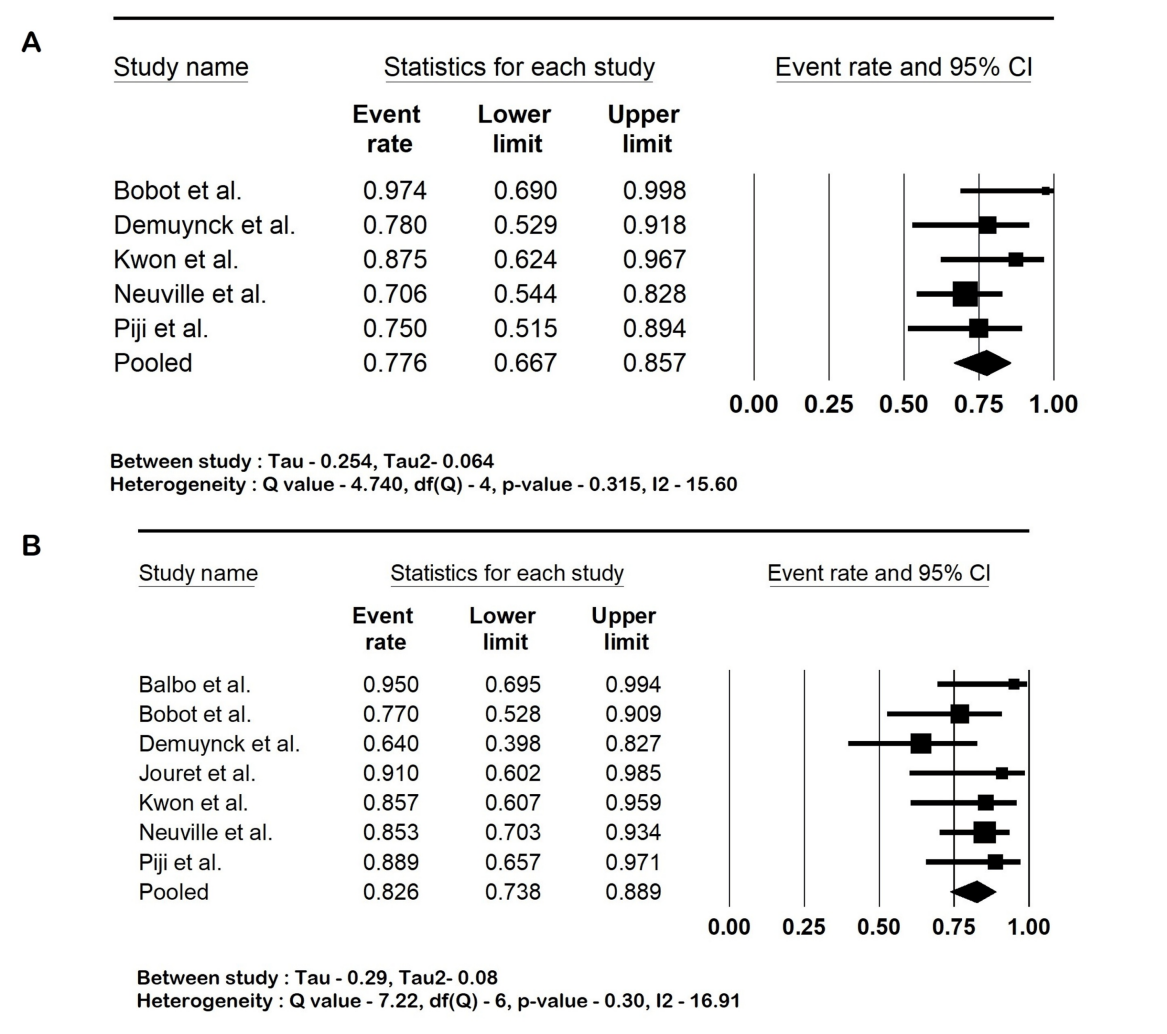
(A) Sensitivity and (B) specificity of 18-fluorodeoxyglucose positron-emission tomography-computed tomography (18F-FDG PET-CT). Balbo et al. [17], Bobot et al. [18], Demuynck et al. [19], Jouret et al. [20], Kwon et al. [21], Neuville et al. [22], and Pijl et al. [23].

**Figure 3:**
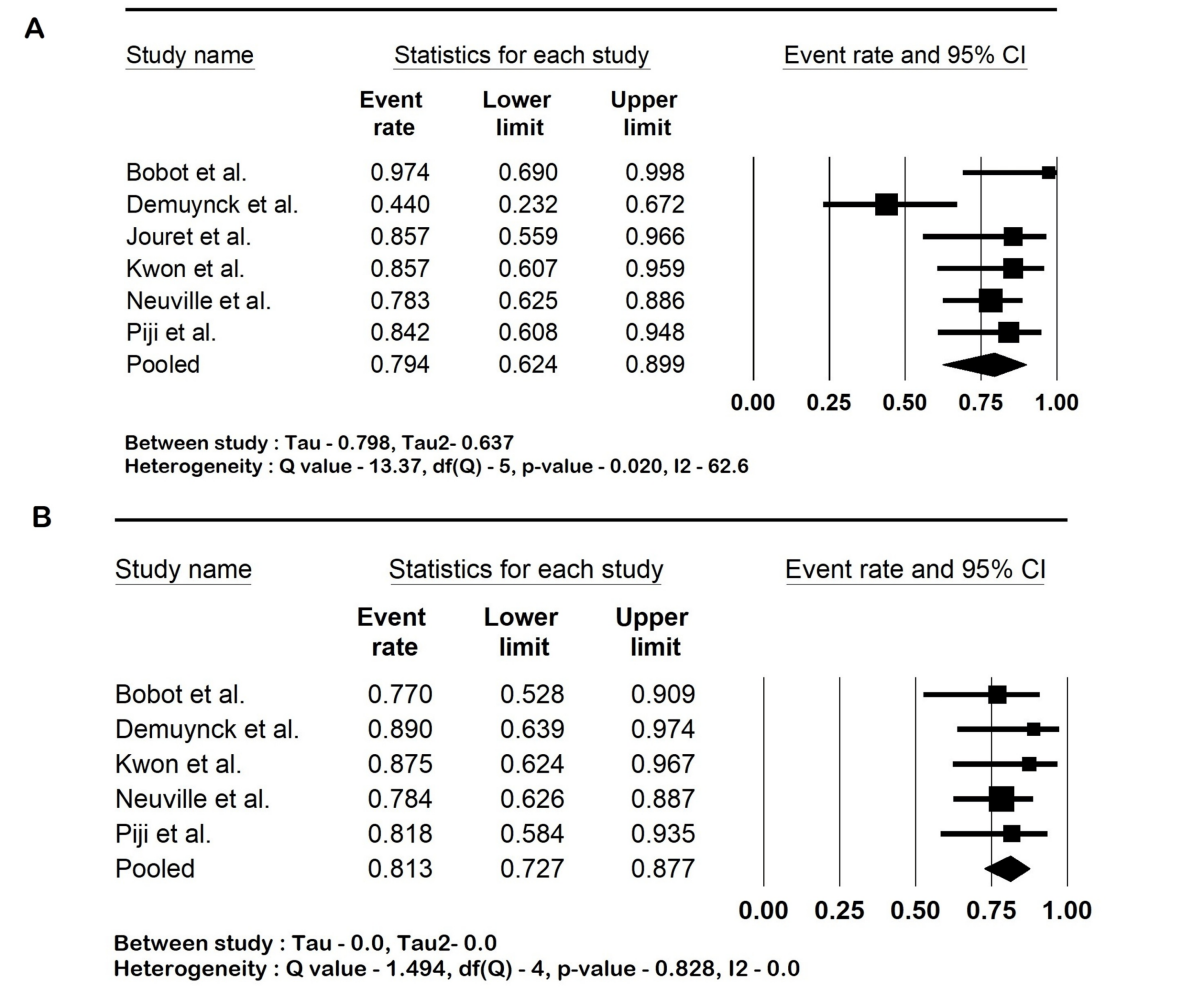
(A) Positive predictive value and (B) negative predictive value of 18-fluorodeoxyglucose positron-emission tomography/computed tomography (18F-FDG PET-CT). Bobot et al. [18], Demuynck et al. [19], Jouret et al. [20], Kwon et al. [21], Neuville et al. [22], and Pijl et al. [23]

**Figure 4:**
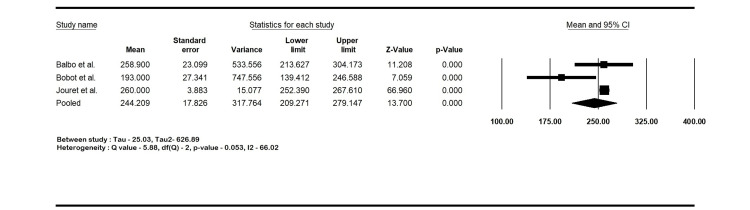
Mean C-reactive protein (CRP) of the patients with cyst infections. Balbo et al. [17], Bobot et al. [18], and Jouret et al. [20]

The pooled sensitivity of ^18^F-FDG PET-CT in diagnosing kidney and hepatic cyst infection was 82.6% (95% CI: 73.8-88.9; I2 16.9%). The specificity was 77.6% (95% CI: 66.7-85.7; I2 15.6%), the PPV was 79.4% (95% CI: 62.4-89.9; I2 62.6%), and the NPV was 81.3% (95% CI: 72.7-87.7; I2 0%). The mean CRP was 244.2 mg/L (95% CI: 209.1-279.1; I2 66%). The forest plots are shown in Figures 2-4.

Sensitivity Analysis

A sensitivity analysis was conducted to ascertain whether any of the included studies had a dominant effect on the outcomes. We systematically removed each study, and the effect size was calculated. The effect sizes of the outcomes did not differ except in the case of PPV, and we noted a considerable difference due to the study by Demuynck et al. [19]. To analyze this further, we removed the study by Demuynck et al., and the pooled PPV was calculated again. The pooled PPV, excluding Demuynck et al., was 83.5% (95% CI: 75.8-89.2). The sensitivity analysis forest plots are shown in the Appendix. An assessment for publication bias was not performed since the total number of studies in the meta-analysis was less than 10.

Quality Assessment and Risk of Bias

**Figure 5:**
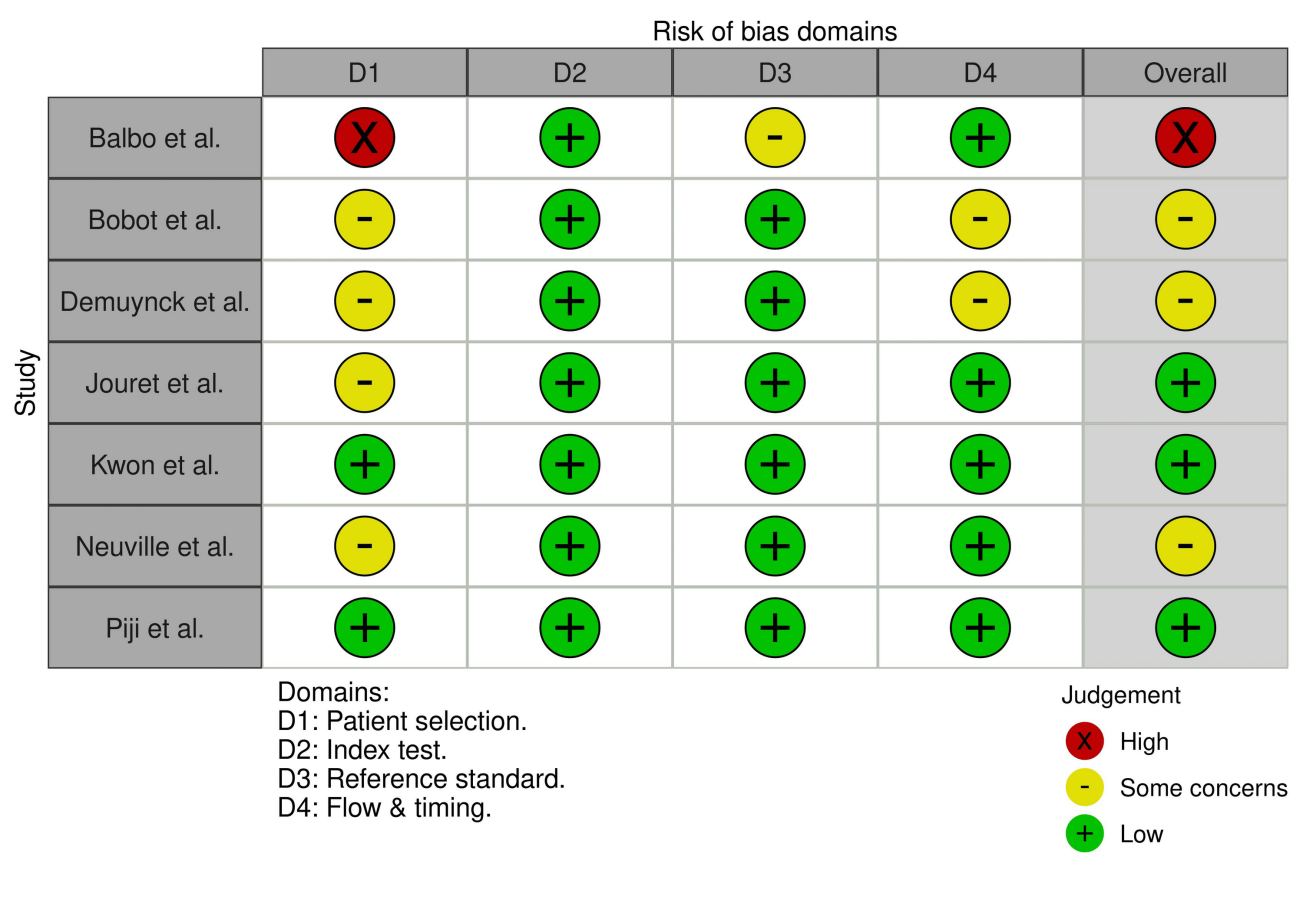
Quality Assessment of Diagnostic Accuracy Studies 2 (QUADAS-2) assessments of included studies. The domains addressing bias are shown, D1: risk of bias due to patient selection; D2: risk of bias due to index test; D3: risk of bias due to reference standard; and D4: risk of bias due to flow and training. Balbo et al. [17], Bobot et al. [18], Demuynck et al. [19], Jouret et al. [20], Kwon et al. [21], Neuville et al. [22], and Pijl et al. [23]

**Figure 6:**
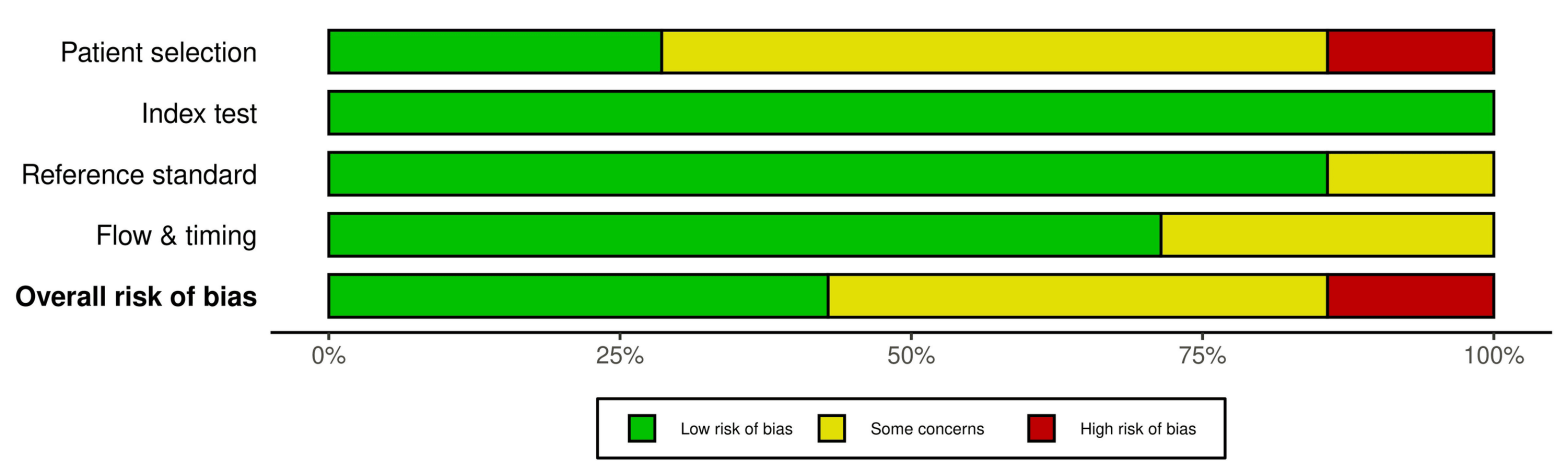
Summary plot of Quality Assessment of Diagnostic Accuracy Studies 2 (QUADAS-2) assessments of all the included studies. Note: This image is the author's own creation.

The quality assessment is summarized in Figures 5, 6.

The risk of bias due to patient selection was considered high in the study by Balbo et al. Also, it was felt that there were some concerns about the reference standard due to the possibility of subjectivity in diagnosing cyst infections based on clinical symptoms and after exclusions [17]. In some studies, the applicability of patient selection criteria was unclear [18-20,22]. There was some missing data on whether all the patients in the cohort underwent ^18^F-FDG PET-CT and the reference standard [18,19].

Heterogeneity

Both the Q statistic and I2 statistics were employed to evaluate heterogeneity. When the Q statistic identified heterogeneity in the analysis, we utilized I2 statistics to quantify its magnitude. Our findings indicate that heterogeneity was low during the estimations of sensitivity, specificity, and NPV since they were <30%. It was substantial during estimations of PPV and CRP effect sizes. Unlike sensitivity and specificity, the prevalence of the disease in the population under study influences PPV, which could explain the substantial heterogeneity in PPV. As the prevalence varies across the studies included in the meta-analysis, this could cause variability in the PPV, leading to substantial heterogeneity.

Discussion

The prevalence of cyst infections among patients with autosomal dominant polycystic kidney disease (ADPKD) is around 8.4% or 0.01 episodes per patient per year [1]. Infections represent the second most common cause of mortality in patients with ADPKD, accounting for about 24% of the deaths; therefore, an early diagnosis is imperative [24]. Renal and hepatic cyst infections in ADPKD present with non-specific symptoms such as fever and abdominal pain, which are difficult to distinguish from cyst hemorrhage [1]. Renal cyst infections can also occur in solitary renal cysts and the presence of multiple renal cysts due to etiologies other than ADPKD. Still, these cases represent only a small proportion [4]. The increased total kidney volume and height-adjusted kidney volume in ADPKD are risk factors for cyst infections [4]. Similarly, urolithiasis and obstructive uropathy are also predisposing factors to cyst infections [25]. Routine tests commonly used, such as ultrasound, are positive in only 13% of the cases, and blood cultures result in an identification of a pathogen in only 49% [3,5]. ^18^F-FDG PET-CT is being used in numerous conditions to aid in diagnosis and has proven utility in diagnosing renal and hepatic cyst infections [26]. During infection and inflammation, tissue perfusion increases, and glucose transporters (GLUTs) are upregulated. Since FDG is a glucose analog, this gets taken up by the GLUT transporters, which is the basis of the test’s highly sensitive nature. It has also been shown that ^18^F-FDG PET-CT has less inter-observer variability than other imaging [27]. Studies have also shown its superiority compared to CT or MRI in diagnosing cyst infections [18,20]. Though no current guidelines exist to establish this modality's role in identifying cyst infections, an international panel of experts in ADPKD, through a Delphi consensus-based survey, recommends that ^18^F-FDG PET-CT should be used in cases of diagnostic uncertainty [5]. 

The typical imaging pattern observed using ^18^F-FDG PET-CT is a localized and elevated radiotracer uptake surrounding the infected cyst. Other uptake patterns of tracer accumulation within the cyst have also been described [4]. These uptake patterns are different in other pathologies involving the kidneys. For instance, a variable uptake pattern is present in renal tumors such as angiomyolipomas, oncocytomas, and renal cell carcinomas. A multifocal uptake pattern is seen in renal leukemias and metastatic lesions [25]. The threshold maximum-standardized uptake value (SUVmax) of 5.0 is used as an aid for a positive test result [4]. A visual four-point scoring scale that uses a scale to quantify the levels of fluorine-18 fluorodeoxyglucose (^18^F-FDG) accumulation within or near the putative has been used to increase the diagnostic yield [19,22]. This has been validated in a cohort of 51 scans. A threshold of 3 or higher is linked to an odds ratio of approximately 13 for cyst infection [19].

Certain biochemical tests also aid in the clinical diagnosis of cyst infection. An elevated CRP >5 mg/dL has been proposed as a diagnostic criterion for the diagnosis [1,6]. Ronsin et al. used ^18^F-FDG PET-CT to diagnose renal cyst infections in renal transplant recipients. Using the diagnostic criteria proposed by Sallée et al., they reported that cyst infections were a risk factor for allograft loss. Also, a CRP cut-off threshold of 50 mg/L was used in their diagnostic criteria. It was also noted that about 25% of the patients in their cohort only had a fever as the presenting symptom, thus highlighting the importance of ^18^F-FDG PET-CT to diagnose these infections, which would have otherwise been missed by conventional imaging [28]. The pooled mean CRP in our study was 244.2 mg/L. Thus, cyst infections in ADPKD are associated with a much greater increase in CRP levels.

In a large systematic review involving cases with definite or likely cyst infection, 49% had a positive cyst aspirate culture, and *Escherichia coli *(*E. coli*) was the pathogen in 50%. Moreover, there was also a 75% rate of failure with initial therapy, which underscores the reason for early diagnosis [3]. The primary differential diagnosis is intracystic hemorrhage. Some studies have shown an increase in the buildup of ^18^F-FDG in both acute and resolving hematomas. ^18^F-FDG PET-CT can aid in distinguishing between renal and hepatic cyst infections and can also be used to assess response to treatment [29]. However, it is unclear if it can differentiate between cyst infections and bleeding [20].

The use of ^18^F-FDG PET-CT has some limitations. Other inflammatory states or cancer could mimic infections [25]. Due to the renal excretion of ^18^F-FDG, a high concentration of the radiotracer could be found in the pyelum of the kidneys, making the diagnosis of concomitant pyelonephritis difficult. Patients with decreased renal function have a decrease in the radiotracer excretion and, thereby, an increase in the background activity compared to the affected lesion. Patients with liver failure may also exhibit a non-localized increase in the uptake. However, it is unclear whether these disproportionate uptakes have any diagnostic consequences in these instances [6].

Our study has a few limitations. First, most of the studies included were retrospective and depended on the thoroughness of the information obtained from medical records. They may contain missing data. Second, our study will also present the inherent biases originally present in the included studies. Third, we report the mean CRP levels during the cyst infection diagnosis. However, CRP levels could be elevated in other conditions associated with inflammation and may not be a reliable marker to detect the infection or denote the severity of the infection. Fourth, since there were a total of only seven studies included in the analyses, we did not perform an analysis to determine publication bias. This could cause a slightly skewed result. It is also possible that the small sample sizes of these studies would affect the overall effect sizes. Finally, the timing of the ^18^F-FDG PET-CT could be different, which might affect the test's positivity. Therefore, caution should be exercised while interpreting the results of meta-analysis. Nonetheless, our study shows the current existing evidence on the utility of ^18^F-FDG PET-CT, which can be applied in clinical practice. Moreover, the sensitivity and specificity reported in our study have low heterogeneity. We also reported the pooled findings of 205 patients from various countries, which is another study's strength.

## Conclusions

The existing literature on patients with ADPKD is sparse, and larger studies to confirm the findings may be difficult to perform due to the disease's low prevalence. Our meta-analysis shows that ^18^F-FDG PET-CT is a highly effective method for enhancing the diagnosis of renal and hepatic cyst infections. This provides more evidence for using ^18^F-FDG PET-CT in difficult clinical settings where other findings may be equivocal.

## Figures and Tables

**Figure 7:**
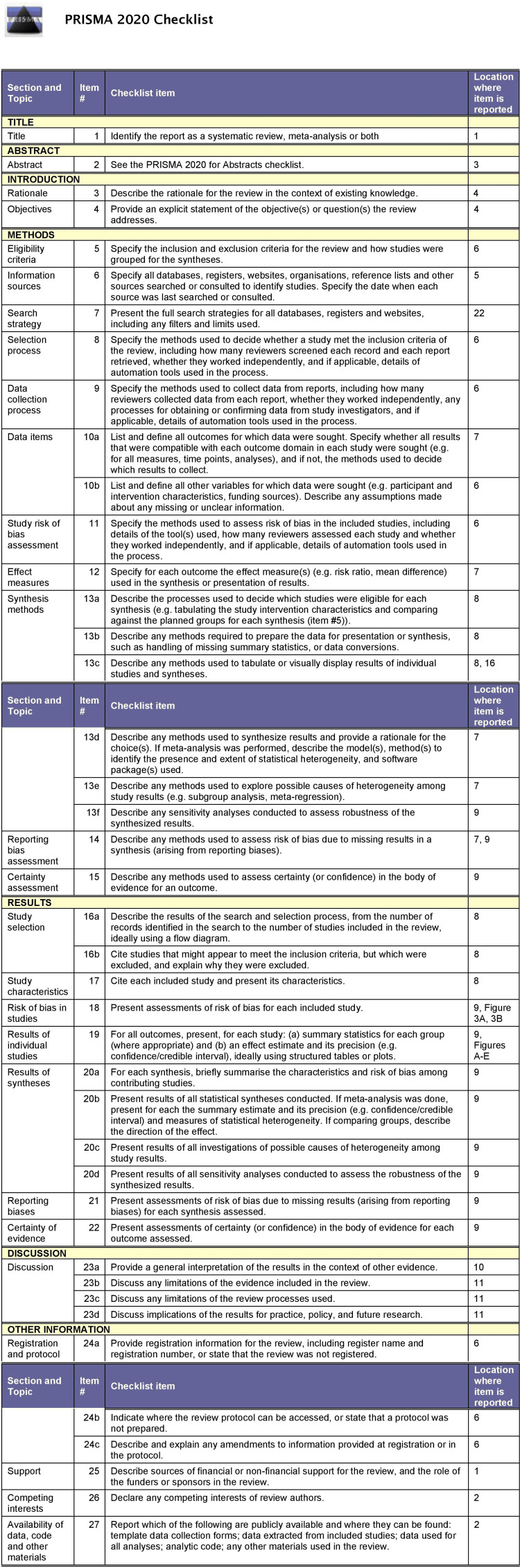
Preferred Reporting Items for Systematic reviews and Meta-Analyses (PRISMA) checklist.

## Appendices

Literature search

A systematic literature review was conducted to evaluate the contemporary literature on ^18^F-FDG PET-CT for detecting cyst infection.

Documentary sources consulted

We carried out a comprehensive literature review using the PubMed, Scopus, Web of Science, and Cochrane library databases.

Search strategy

For a comprehensive search, suitable medical subject headings (MeSH) and index terms were identified to be included in the search term. Index and MeSH terms are controlled vocabularies used to standardize literature indexing, allowing systematic and accurate retrieval of articles from databases.

The query and the search results for each database are as follows:

1. PubMed:

("PET" (Title/Abstract) OR "Positron-emission tomography" (Title/Abstract) OR "CT" (Title/Abstract) OR "computed tomography"(Title/Abstract)) AND ("Autosomal dominant polycystic kidney disease" (Title/Abstract) OR "ADPKD"(Title/Abstract) OR "polycystic kidney disease"(Title/Abstract) OR "renal cyst infection"(Title/Abstract) OR "kidney cyst infection"(Title/Abstract) OR "liver cyst infection"(Title/Abstract) OR "hepatic cyst infection"(Title/Abstract)).

Results 524

2. Scopus:

(TITLE-ABS ("PET" OR "Positron-emission tomography" OR "CT" OR "computed tomography")) AND (TITLE-ABS("Autosomal dominant polycystic kidney disease" OR "ADPKD" OR "polycystic kidney disease" OR "renal cyst infection" OR "kidney cyst infection" OR "liver cyst infection" OR "hepatic cyst infection"))

Results: 544

3. Web of Science (WOS):

TS=("PET" OR "Positron-emission tomography" OR "CT" OR "computed tomography") AND TS=("Autosomal dominant polycystic kidney disease" OR "ADPKD" OR "polycystic kidney disease" OR "renal cyst infection" OR "kidney cyst infection" OR "liver cyst infection" OR "hepatic cyst infection")

Results: 569

4. Cochrane Library:

("PET" OR "Positron-emission tomography" OR "CT" OR "computed tomography") AND ("ADPKD" OR "polycystic kidney disease" OR "renal cyst infection" OR "kidney cyst infection" OR "liver cyst infection" OR "hepatic cyst infection")

Results: 63

Total: 1700

Duplicates: 649

Total after removing duplicates: 1051

## References

[1] Sallée M, Rafat C, Zahar JR, Paulmier B, Grünfeld JP, Knebelmann B, Fakhouri F (2009). Cyst infections in patients with autosomal dominant polycystic kidney disease. Clin J Am Soc Nephrol.

[2] Suwabe T, Ubara Y, Higa Y (2009). Infected hepatic and renal cysts: differential impact on outcome in autosomal dominant polycystic kidney disease. Nephron Clin Pract.

[3] Lantinga MA, Casteleijn NF, Geudens A (2017). Management of renal cyst infection in patients with autosomal dominant polycystic kidney disease: a systematic review. Nephrol Dial Transplant.

[4] Ueda CE, Ono CR (2018). Role of 18F-FDG PET/CT in renal cyst infection. Curr Radiol Rep.

[5] Lantinga MA, Darding AJ, de Sévaux RG (2016). International multi-specialty Delphi survey: identification of diagnostic criteria for hepatic and renal cyst infection. Nephron.

[6] Pijl JP, Nienhuis PH, Kwee TC, Glaudemans AW, Slart RH, Gormsen LC (2021). Limitations and Pitfalls of FDG-PET/CT in infection and inflammation. Semin Nucl Med.

[7] Stroup DF, Berlin JA, Morton SC (2000). Meta-analysis of observational studies in epidemiology: a proposal for reporting. JAMA.

[8] Page MJ, McKenzie JE, Bossuyt PM (2021). The PRISMA 2020 statement: an updated guideline for reporting systematic reviews. BMJ.

[9] Whiting P, Rutjes AW, Reitsma JB, Bossuyt PM, Kleijnen J (2003). The development of QUADAS: a tool for the quality assessment of studies of diagnostic accuracy included in systematic reviews. BMC Med Res Methodol.

[10] McGuinness LA, Higgins JPT (2021). Risk-of-bias VISualization (robvis): an R package and Shiny web app for visualizing risk-of-bias assessments. Res Synth Methods.

[11] Borenstein M, Hedges LV, Higgins JP, Rothstein HR (2010). A basic introduction to fixed-effect and random-effects models for meta-analysis. Res Synth Methods.

[12] DerSimonian R, Laird N (1986). Meta-analysis in clinical trials. Control Clin Trials.

[13] Sutton AJ, Abrams KR, Jones DR, Jones DR, Sheldon TA, Song F (2000). Methods for Meta-Analysis in Medical Research.

[14] Biggerstaff BJ, Tweedie RL (1997). Incorporating variability in estimates of heterogeneity in the random effects model in meta-analysis. Stat Med.

[15] Higgins JP, Thompson SG, Deeks JJ, Altman DG (2003). Measuring inconsistency in meta-analyses. BMJ.

[16] (2024). Comprehensive Meta-Analysis software, version 4. https://meta-analysis.com/.

[17] Balbo BE, Sapienza MT, Ono CR, Jayanthi SK, Dettoni JB, Castro I, Onuchic LF (2014). Cyst infection in hospital-admitted autosomal dominant polycystic kidney disease patients is predominantly multifocal and associated with kidney and liver volume. Braz J Med Biol Res.

[18] Bobot M, Ghez C, Gondouin B (2016). Diagnostic performance of [(18)F]fluorodeoxyglucose positron emission tomography-computed tomography in cyst infection in patients with autosomal dominant polycystic kidney disease. Clin Microbiol Infect.

[19] Demuynck S, Lovinfosse P, Seidel L (2023). Standardized 4-point scoring scale of [(18)F]-FDG PET/CT imaging helps in the diagnosis of renal and hepatic cyst infections in patients with autosomal dominant polycystic kidney disease: a validation cohort. Clin Kidney J.

[20] Jouret F, Lhommel R, Beguin C, Devuyst O, Pirson Y, Hassoun Z, Kanaan N (2011). Positron-emission computed tomography in cyst infection diagnosis in patients with autosomal dominant polycystic kidney disease. Clin J Am Soc Nephrol.

[21] Kwon HW, Lee HY, Hwang YH, Park HC, Ahn C, Kang KW (2016). Diagnostic performance of 18F-FDG-labeled white blood cell PET/CT for cyst infection in patients with autosomal dominant polycystic kidney disease: a prospective study. Nucl Med Commun.

[22] Neuville MF, Lovinfosse P, Jadoul A, Thys M, Seidel L, Hustinx R, Jouret F (2021). The use of a visual 4-point scoring scale improves the yield of (18)F-FDG PET-CT imaging in the diagnosis of renal and hepatic cyst infection in patients with autosomal dominant polycystic kidney disease. Eur J Nucl Med Mol Imaging.

[23] Pijl JP, Glaudemans AW, Slart RH, Kwee TC (2018). (18)F-FDG PET/CT in autosomal dominant polycystic kidney disease patients with suspected cyst infection. J Nucl Med.

[24] Fick GM, Johnson AM, Hammond WS, Gabow PA (1995). Causes of death in autosomal dominant polycystic kidney disease. J Am Soc Nephrol.

[25] Kochhar R, Brown RK, Wong CO, Dunnick NR, Frey KA, Manoharan P (2010). Role of FDG PET/CT in imaging of renal lesions. J Med Imaging Radiat Oncol.

[26] Casali M, Lauri C, Altini C (2021). State of the art of (18)F-FDG PET/CT application in inflammation and infection: a guide for image acquisition and interpretation. Clin Transl Imaging.

[27] Vaidyanathan S, Patel CN, Scarsbrook AF, Chowdhury FU (2015). FDG PET/CT in infection and inflammation-current and emerging clinical applications. Clin Radiol.

[28] Ronsin C, Chaba A, Suchanek O (2022). Incidence, risk factors and outcomes of kidney and liver cyst infection in kidney transplant recipient with ADPKD. Kidney Int Rep.

[29] Lantinga MA, Drenth JP, Gevers TJ (2015). Diagnostic criteria in renal and hepatic cyst infection. Nephrol Dial Transplant.

